# Genetic Variant in Leptin rs7799039 Is Associated with Total Body Mineral Estimates, but Not with Fat Mass, in Young Healthy Adults

**DOI:** 10.3390/nu18030481

**Published:** 2026-02-01

**Authors:** Darina Falbová, Lenka Vorobeľová

**Affiliations:** Department of Anthropology, Faculty of Natural Sciences, Comenius University in Bratislava, 82105 Bratislava, Slovakia; lenka.vorobelova@uniba.sk

**Keywords:** young adults, bioimpedance analysis, leptin, G-2548A, genetic variant

## Abstract

Background/Objectives: The aim of this study was to investigate the association between the rs7799039 variant in the leptin (*LEP*) gene and specific parameters of body composition in young healthy Slovak adults using bioelectrical impedance analysis. Methods: We assessed 467 young adults aged 18 to 30 years with an average age of 22.55 ± 2.56 years. Genotyping of SNP LEP G-2548A (rs7799039) was performed by polymerase chain reaction-restriction fragment length polymorphism (PCR-RFLP) and body composition was assessed by bioelectrical impedance analysis (InBody 770). Results: Our results showed that the *LEP* rs7799039 variant was associated with total body mineral levels in women. The mean values of total body minerals (kg) were higher in *LEP* AA carriers than in carriers of the G allele (3.26 ± 0.52 kg compared to 3.09 ± 0.36 kg; *p* = 0.014). In addition, linear regression analysis showed statistically significant associations of the *LEP* gene rs7799039, vitamin D intake, body mass index (BMI) and height on total body mineral content in women (*p* < 0.05). The presence of the *LEP* AA genotype, reported vitamin D intake and higher BMI and height values were positively associated with higher total body mineral content. No association was found between the *LEP* rs7799039 variant and BMI, fat mass (FM) or FM distribution across body segments. Conclusions: Our data suggest that the rs7799039 variant in the *LEP* gene may be associated with small differences in total body mineral content in young adult women. These findings should be interpreted as exploratory associations, rather than evidence of biological specificity or an independent genetic effect.

## 1. Introduction

Leptin (*LEP*) is a cytokine that is mainly synthesised in white adipose tissue (16 kDa peptide) and plays a central role in the regulation of body weight and energy homeostasis. As a key mediator in the signalling pathway between adipose tissue and the brain, *LEP* modulates feelings of hunger and satiety and thus is involved in the regulation of energy intake and energy expenditure [1,2]. In addition to its known effects on energy balance, *LEP* has been suggested to be involved in mineral metabolism via direct and indirect mechanisms. It interacts with bone cells, particularly osteoblasts and osteoclasts, and has been associated with processes related to bone remodelling and mineral density. Moreover, LEP-related signalling through the hypothalamic–pituitary axis and the sympathetic nervous system has been proposed to contribute to calcium and phosphate homeostasis, highlighting its broader systemic relevance [3]. These pathways have been discussed as potential links to mineral-related outcomes, including overall mineral distribution in the body.

The gene for *LEP* is located on chromosome 7q31.3, encodes a 3.5 kb cDNA and consists of three exons and two introns [1]. Genetic variants in the *LEP* gene have been studied in relation to adipose tissue metabolism, body mass regulation, lipid and glucose metabolism, and endocrine and cardiovascular functions. Consequently, investigation of LEP gene variants may contribute to a better understanding of inter-individual variability in obesity and related metabolic disorders.

To date, several studies have examined variants in the *LEP* gene and their association with obesity risk in different ethnic populations. However, the results have often been contradictory [4,5,6,7]. The most extensively studied variant, rs7799039 (G-2548A), has been examined in relation to metabolic effects, obesity and eating behaviour, including eating habits and satiety. This SNP is a guanine-to-adenine substitution in the promoter region and has been suggested to influence transcriptional activity and LEP expression [8]. The A allele has been associated with higher LEP production and secretion compared to the G allele [9]. Variability in LEP expression has been linked to metabolic parameters and satiety regulation [10,11]. Nevertheless, studies investigating rs7799039 have reported conflicting results regarding its association with body weight and body composition [12,13,14,15]. While some studies indicated that the G allele was associated with higher anthropometric measures or obesity risk [12,16], others reported associations with the A allele [8,17].

Assessment of body composition has become increasingly important in obesity research, as it complements traditional measures such as body mass index (BMI), which may not fully capture variability in body fat distribution and related metabolic risk. Detailed body composition analysis provides additional insight into metabolic health, particularly in young adult populations [18].

Given these considerations, the present cross-sectional study aims to examine the association between the *LEP* rs7799039 variant and selected parameters of body composition, including fat mass- and fat-free mass-related indices across different body segments, as well as total body mineral estimates, in young healthy adults from Slovakia.

## 2. Materials and Methods

### 2.1. Participants

The study population consisted of 467 Slovak young adults aged 18 to 30 years, with an overall mean age of 22.55 ± 2.56 years. Of these, 264 were women (mean age 22.32 ± 2.57) and 203 were men (mean age 22.84 ± 2.51). Most participants (n = 406) were recruited before the COVID-19 pandemic, between February 2019 and March 2020, while a smaller group (n = 61) was enrolled after the pandemic in April 2023. Recruitment was voluntary, and all assessments were conducted at the Biomedical Laboratory of the Department of Anthropology, Comenius University, Bratislava, Slovakia. Participants were recruited through various channels, including university email notifications, online student forums, and flyers at Comenius University. Announcements were made during lectures to inform students about the research opportunity. Recruitment occurred on a rolling basis, ensuring a diverse sample of university students and graduates. Participants were informed that participation was voluntary and confidential, and that they could withdraw at any time without consequence. Participant information, including name, date of birth, and sex, was recorded, and each individual was assigned a unique identification number to maintain anonymity. All participants provided written informed consent prior to participation, in accordance with the principles of the Declaration of Helsinki. The study procedures were reviewed and approved by the Ethics Committee of the Faculty of Natural Sciences at Comenius University (approval number ECH19021). Only participants who provided written consent were included in the study. Inclusion criteria required participants to be Slovak university students or graduates of European origin, aged 18 to 30 years, and without acute medical conditions. Participants were excluded if they had any of the following conditions: thyroiditis, Crohn’s disease, liver disorders, type 1 diabetes, epilepsy, or any form of oncological disease. The final study sample included only individuals who met all specified inclusion and exclusion criteria. Of the entire sample, 310 participants provided all required data: anthropometric, body composition, and genetic data. The remaining participants (n = 157) did not provide adequate information for at least one of these factors.

### 2.2. Questionnaire

Baseline characteristics and sociodemographic information were collected using a Slovak translation of a standardised and validated questionnaire based on the WHO STEPwise approach to surveillance (version 3.2, 2014) [19]. As the information depended on participants’ own perceptions, memories, and judgements, the resulting data were inherently subjective. Lifestyle habits were recorded through a combination of self-administered responses and structured interviews. Smoking status, alcohol use, physical activity, and intake of calcium and vitamin D were initially assessed with simple yes/no questions. To obtain more detailed insights into smoking and drinking behaviours, participants were further asked to indicate the frequency of these habits using seven categories: daily, 5–6 days per week, 3–4 days per week, 1–2 days per week, 1–3 days per month, less than once per month, or never. Physical activity was then categorised into three levels according to WHO guidelines for adults aged 18–64 [20]. The lowest category (tier 0) included participants who did not meet the recommended minimum, engaging in only incidental movement such as walking to work, and performing less than 300 min of moderate or 150 min of vigorous activity per week. The intermediate group (tier 1) comprised individuals achieving 150–300 min of moderate or 75–150 min of vigorous activity per week, or exceeding these levels but exercising only one or two days per week. The highest category (tier 2) included participants regularly surpassing 300 min of moderate or 150 min of vigorous activity weekly, typically exercising around three times per week. These questions were taken from Step 1 of the WHO STEPS instrument, which differentiates between moderate and vigorous intensity activities.

### 2.3. Body Composition Measurement

Body composition was assessed using the InBody 770 analyser (Biospace Co., Seoul, Republic of Korea), which operates on the principle of segmental multi-frequency bioelectrical impedance analysis. This method estimates body composition by transmitting low-intensity electrical currents through the body and evaluating tissue-specific resistance. The device provides measurements of lean body mass (LBM) and fat mass (FM) for the whole body, trunk, and arms, expressed in both kilograms and percentages. It also determines the fat-free mass index (FFMI), fat mass index (FMI), phase angle, visceral fat area (cm^2^), percent body fat (PBF), total body minerals, body cell mass (BCM), skeletal muscle mass (SMM), and fat-free mass (FFM) in kilograms for both sexes. Validation studies comparing the InBody 770 with dual-energy X-ray absorptiometry (DXA) have demonstrated a 98% correlation between the two techniques [21]. To ensure measurement reliability, all assessments were conducted under standardised conditions. Participants were tested in the morning after refraining from physical activity for at least eight hours and avoiding substantial food or water intake for three hours prior to measurement. During the procedure, individuals stood barefoot on the scale’s electrode platform and held the hand electrodes at approximately a 15° angle to prevent arm-to-torso contact. Body weight was recorded to the nearest 0.1 kg using the device’s integrated scale.

### 2.4. Anthropometric Measurement

Anthropometric measurements were obtained by trained anthropologists following internationally recognised and standardised procedures [22]. Body height was assessed using an anthropometer (Sieber & Hegner; Zürich, Switzerland) with a precision of 0.5 cm. Participants were measured in an upright posture, barefoot, with their feet together. Waist and hip circumferences were determined with a tape measure (Seca, Hamburg, Germany) while participants stood upright, abdomen relaxed, feet together, and arms folded across the chest. For waist measurements, the examiner stood facing the participant, placed the tape around the narrowest section of the abdomen without compressing the soft tissue, and took the reading from the right side of the body at the end of a normal exhalation. Hip circumference was measured in the same stance, with the tape applied around the widest part of the buttocks while the examiner remained on the right side. BMI was calculated as body weight (kg) divided by height squared (m^2^), and the waist-to-hip ratio (WHR) was calculated as waist circumference divided by hip circumference.

### 2.5. Buccal Swab Sampling

The probands did not eat, drink, smoke, chew gum or perform oral hygiene for at least one hour before the swab was taken. Swabs were performed by rubbing the sticky end for about 30 s on the gum and on the inside of the cheek, allowing us to collect the flaking cells of the oral mucosa; they were then placed in a special test tube, without liquid or culture medium, and stored in the refrigerator before the extraction procedure.

### 2.6. DNA Extraction and Genotyping Description

DNA was extracted from saliva samples using the Buccal Swab DNA Extraction Kit (Spin-column; (Amplia, Bratislava, Slovakia)); and the *LEP* gene rs7799039 SNP variant was detected by polymerase chain reaction-restriction fragment length polymorphism (PCR-RFLP).

Genomic DNA (1 μL) was incubated in a 20 μL solution containing 13.9 μL H_2_O, 2.0 μL 10×Taq Buffer with KCl, 1.5 μL MgCl_2_ (25 mM), 0.2 μL dNTPMix (10 mM), 0.3 μL (50 pmol) forward primer (5′-TTT CCT GTA ATT TTC CCG TGA G-3′), 0.3 μL (50 pmol) reverse primer (5′-AAA GCA AAG ACA GGC ATA AAA A-3′), and 0.1 μL Taq DNA Polymerase (recombinant). The PCR mix was moved into a thermal cycler (VWR International bvba, Leuven, Belgium) and amplified by heating at 94 °C for 4 min followed by 33 cycles of 94 °C for 1 min, 58 °C for 1.30 min, 72 °C for 2 min and final incubation at 72 °C for 2 min. Amplicons (242 bp) were then digested for 5 h by *HhaI* restriction enzyme, and the fragments were separated by electrophoresis on 3% agarose gel with stain (ethidium bromide). The 242 bp PCR product (AA) was digested into 181 bp (GG) and 61 bp (GA) fragments in the presence of a G nucleotide (polymorphic variant) but not in its absence.

### 2.7. Statistical Analysis

All statistical analyses were conducted using SPSS software (version 24, IBM Corp., Armonk, NY, USA) for Windows, and results with *p*-values below 0.05 were considered statistically significant. The study design was cross-sectional. The linear regression model sample size was calculated a priori considering the following parameter values: effect size = 0.3, α = 0.05, and power = 0.80. This calculation indicated that a total of 91 participants would be required. The sample size was determined using the novel approach proposed by Guan et al. [23]. This calculation was intended to ensure adequate power for regression modelling rather than to estimate the magnitude of observed effects. For each sex group, descriptive statistics were calculated, and data normality was evaluated using the Kolmogorov–Smirnov test. Genotype and allele distribution was analyzed using the χ^2^ test. All genetic association analyses were performed separately in men and women to allow sex-specific evaluation of genotype–phenotype relationships. An independent samples t-test was applied to compare quantitative variables between groups, whereas the Mann–Whitney U test was used for non-normally distributed data within in both sexes. Furthermore, due to reduce the increased risk of a type I error, the Holm-Bonferroni correction was applied. The *p*-value was compared to an adjusted threshold, calculated as *α*/m-*i*+1, where *α* = 0.05, m is the total number of tests, and *i* is the rank of the test. Only those tests with *p*-values smaller than their corresponding adjusted thresholds were considered statistically significant. The Holm–Bonferroni correction was applied separately within each sex-stratified set of univariate comparisons, thereby controlling the family-wise error rate within biologically comparable outcome groups. Forward stepwise linear regression analysis was performed to examine associations between total body mineral content (kg) and the following independent variables: smoking status, alcohol consumption, calcium and vitamin D intake, physical activity, *LEP* rs7799039 genotype (AA vs. GG + GA), BMI, and height. Variables were entered into the model using a forward selection procedure, and predictors with *p*-values < 0.05 were retained in the final model. Multicollinearity was assessed using variance inflation factors (VIFs), with all VIF values < 2.0, indicating no relevant collinearity among predictors. The forward stepwise approach was applied in an exploratory manner to identify statistically relevant covariates, without implying causal relationships.

## 3. Results

The baseline characteristics of the entire sample are listed in Table 1. These characteristics are divided into demographic characteristics such as sex and age, various lifestyle variables such as smoking status, frequency of alcohol consumption, physical activity, calcium and vitamin D intake, and genetic data.

The study sample consisted of young adults aged 19 to 29 years, with an average age of 22.35 ± 2.28 years, including 157 women and 153 men. Most of them were non-smokers (80.97%), consumed alcohol 1 to 3 days per month (34.52%), engaged in less than 300 min of moderate or 150 min of vigorous physical activity per week, and participated in occasional activities such as walking to work (53.87%). Only 11.61% of participants reported calcium intake and only 16.77% reported vitamin D intake. The genotype distribution of the *LEP* variant was 14.84% (n = 46), 54.19% (n = 168) and 30.97% (n = 96) of participants for the GG, GA and AA genotypes, respectively. The distribution corresponded to the Hardy–Weinberg equilibrium (*p* = 0.05). In addition, the frequency of the A allele was found to be higher than that of the G allele (0.58 vs. 0.42).

Table 2 compares the mean values of selected anthropometric parameters and body composition according to *LEP* rs7799039 genotypes (GG + GA vs. AA) separately for women and men.

Only associations that remained statistically significant after Holm–Bonferroni correction are reported and discussed. In both study groups, no statistically significant differences in anthropometric variables were observed depending on the *LEP* rs7799039 genotype. *LEP* rs7799039 genotypes showed a statistically significant association with total body minerals (kg) (*p* = 0.014) in women. The mean values of total body minerals (kg) were higher in *LEP* AA carriers than in carriers of the G allele (3.26 ± 0.52 kg compared to 3.09 ± 0.36 kg). Although FFMI showed higher mean values in AA carriers in unadjusted analyses (15.93 ± 1.34 vs. 15.56 ± 1.30), this difference did not remain significant after multivariable adjustment.

The forward method of linear regression analysis was used to examine independent associations between selected dietary and lifestyle factors and total body minerals (kg). The Durbin–Watson test indicated no evidence of autocorrelation. In women (Table 3), the predictors *LEP* gene rs7799039 genotype, vitamin D intake, BMI, and height showed statistically significant associations with total body mineral content (kg). Positive values of the B coefficient indicate that the *LEP* AA genotype, reported vitamin D intake, and higher BMI and height were associated with higher total body mineral estimates in women.

## 4. Discussion

In this cross-sectional study, the genetic variant in the *LEP* gene rs7799039 was analysed in young adults from Slovakia to investigate its association with selected body composition parameters.

The genotype distribution for the G-2548A variant was GG 14.84%, GA 54.19% and AA 30.97%, with a prevalence of the A allele of 58.00%. The distribution of the A allele in our study is in good agreement with other European populations. For example, with the results in southern Europe, such as Spain, where the prevalence of the A allele is 55% [24]. In contrast, southeastern European populations, such as the Serbian [25] and Romanian [26] populations, tend to have slightly lower A allele frequencies, often around 40–47%, which may reflect regional genetic heterogeneity shaped by historical and environmental factors.

In the present study, no significant association was observed between the *LEP* rs7799039 variant and BMI, FM, or FM distribution across body segments. These findings are consistent with those of García et al. [9], who reported no association between *LEP* rs7799039 and body composition parameters in young adults from western Mexico, suggesting that this variant may not represent a robust genetic marker of adiposity in all populations. Similarly, studies conducted in Turkey [27] and Romania [28] reported no association between this *LEP* variant and BMI. In contrast, several investigations have identified associations between rs7799039 and obesity-related traits in specific populations, including Brazilians [15], Tunisians [17], and Taiwanese aborigines [29]. Jiménez-Osorio et al. [30] further demonstrated that the presence of the AA or AG genotype was associated with obesity in Mexican adults, while Raskiliene et al. [31] reported that the GG genotype was associated with higher BMI, waist circumference, and visceral fat levels exclusively in Lithuanian men. Together, these findings highlight the population- and sex-specific nature of associations between *LEP* genetic variation and adiposity-related traits.

The *LEP* gene variant rs7799039 showed a statistically significant association with total body minerals (kg) only in women (*p* = 0.014), with *LEP* AA carriers having a higher amount of minerals than the G allele carriers (3.26 ± 0.52 kg vs. 3.09 ± 0.36 kg). Although the observed between-genotype difference in total body minerals was small, the aim of the present analysis was not to define clinically relevant thresholds, but to explore genotype-related variation at the population level. Importantly, the magnitude of this association was small and should not be interpreted as evidence of a biologically independent genetic effect, but rather as an exploratory observation that requires confirmation using more precise phenotyping methods and independent population-based studies. To our knowledge, few population-based studies have examined the association between *LEP* rs7799039 and total body mineral estimates in adults.

Although FFMI differed between genotype groups in unadjusted analyses, it was not independently associated with genotype in multivariable models after adjustment for relevant covariates. In contrast, total body minerals showed a significant association with *LEP* rs7799039 in the forward regression analyses. FFMI and total body minerals represent related but distinct components of fat-free mass and are derived from the same BIA framework. These findings suggest that genotype-related variation in body composition, if present, may be more readily detected in the mineral component of fat-free mass than in height-indexed fat-free mass measures. However, given that FFMI and total body mineral estimates are derived from the same bioelectrical impedance framework, this pattern may also reflect measurement-related interdependence among closely related body composition indicators rather than true biological specificity.

Previous studies have demonstrated that the rs7799039 SNP is associated with circulating leptin concentrations, with AA genotype carriers generally exhibiting higher serum leptin levels than individuals carrying the AG or GG genotype [1,4,25,32]. Hoffstedt et al. [1] reported that the AA genotype is associated with increased *LEP* gene expression and leptin secretion from adipose tissue. Given the central role of leptin in regulating energy balance, glucose homeostasis, neuroendocrine function, immune responses, and metabolic activity, it is biologically plausible that leptin-related pathways may be related to mineral metabolism, although such links remain indirect and cannot be verified within the present study [33,34,35].

This interpretation is supported by findings from Alghadir et al. [36], who reported positive associations between mineral elements and circulating leptin concentrations. Moreover, evidence summarized in recent review articles indicates that LEP participates in the regulation of bone remodelling processes, and that bone tissue, as the primary mineral reservoir in the human body, contributes substantially to total body mineral content [37,38]. Previous studies have also reported positive associations between leptin and bone mineral density in premenopausal women [39,40], as well as associations with bone area in younger populations, particularly adolescent girls, which has been interpreted as consistent with a potential role in periosteal expansion [41]. Conversely, Blum et al. [42] observed negative associations between leptin concentrations and bone mass in premenopausal women of European origin, underscoring the complexity of leptin–bone interactions.

Based on the present findings, variation in the *LEP* gene was weakly associated with differences in total body mineral estimates, potentially reflecting broader leptin-related pathways involved in energy metabolism and body composition regulation, rather than direct nutritional effects. Given the small effect size and the inherent measurement variability of bioelectrical impedance-derived mineral estimates, these findings should be interpreted with particular caution and regarded as exploratory rather than confirmatory. Importantly, these findings are interpreted as associative rather than causal, as the underlying biological mechanisms cannot be established within the cross-sectional design of this study.

Sex-specific differences in leptin physiology provide a biologically plausible context for the observed associations. Women are known to exhibit higher circulating leptin levels than men, even after adjustment for fat mass [43,44], and this difference is further amplified in the presence of obesity [34]. In the present study, genotype-related associations with total body minerals were observed in women, whereas no statistically significant associations were detected in men. Accordingly, the present findings should not be interpreted as evidence of a women-specific genetic effect, but rather as sex-stratified associations observed within the constraints of this cohort.

However, given the cross-sectional design and the limited sample size of the male subgroup, the absence of statistically significant findings in men should not be interpreted as evidence of a true sex-specific effect. Formal genotype-by-sex interaction testing was not a primary objective of this study and was therefore not performed. Future studies with larger, balanced samples are warranted to directly assess potential sex differences in the magnitude of genotype effects.

Behavioural pathways have been discussed in the literature as a potential indirect link between *LEP* genotype and mineral status. The study by Andreoli et al. [45] provides context for such hypotheses, as it reported that the *LEP* rs7799039 variant has been associated with differences in responses to environmental factors, including eating behaviour (food preferences, food intake, snacking behaviour and portion sizes), sleep patterns, and stress-related experiences, particularly in young adults [9,45,46]. There are relatively few studies investigating the association between *LEP* rs7799039 and food intake in adult populations. For example, the GG genotype of rs7799039 was associated with higher scores in food avoidance appetitive traits (emotional undereating and food fussiness) in Mexican young adults [9]. A case-control study conducted in Tunisia reported that adults carrying the AA genotype exhibited higher reported daily energy intake (*p* = 0.048; GG: 2853 ± 1215 kcal; GA: 2889 ± 1277 kcal; AA: 3431 ± 1609 kcal) [11]. Although such genotype-related behavioural differences have been proposed as potential contributors to variation in dietary intake, this hypothesis cannot be directly evaluated within the present study and therefore remains speculative.

Finally, Lari et al. [47] demonstrated associations between rs7799039, leptin concentrations, and vitamin D levels in healthy individuals. Given the established role of vitamin D in calcium and phosphorus metabolism [48], this pathway represents an additional biologically plausible mechanism linking *LEP* genetic variation to mineral-related outcomes, warranting further investigation in studies incorporating biochemical and dietary assessments.

Our study has several limitations that should be considered when interpreting the findings. First, the number of genotyped participants was relatively small; therefore, replication of these results in independent cohorts with larger sample sizes would be valuable to further support the observed associations. Second, the study sample consisted exclusively of Slovak individuals of European origin, which limits the generalisability of the findings to other populations. Although the study population was relatively homogeneous, adjustment for fine-scale population structure using genome-wide principal components was not possible, as SNP array data were not available. Information on birthplace or more detailed geographic origin was not available and therefore could not be used as an alternative proxy for population structure.

Third, environmental and lifestyle variables, including physical activity, alcohol intake, and consumption of calcium and vitamin D, were assessed using self-reported questionnaires, which may be subject to reporting bias. Calcium and vitamin D intake were assessed using binary (yes/no) variables, which do not allow quantitative evaluation of intake levels, dosage, or nutritional adequacy. In addition, the study did not include detailed dietary records, quantitative assessment of nutrient intake, information on supplementation, or biochemical measures of calcium and vitamin D status. Consequently, the relative contribution of overall dietary patterns versus specific nutritional factors could not be fully disentangled. Given the small magnitude of the observed association, the findings should be interpreted as exploratory and require confirmation using more precise phenotyping methods. In addition to inherent measurement variability, the influence of random variation cannot be fully excluded, particularly given the exploratory nature of the analyses and the small magnitude of the observed association.

Finally, circulating leptin concentrations were not measured. As a result, the present study could not directly evaluate whether the observed genotype–phenotype associations were mediated through differences in leptin secretion or signaling. Future studies incorporating biochemical markers, more detailed dietary assessment, and larger genetically characterised cohorts will be important to clarify the biological pathways underlying these associations.

## 5. Conclusions

To conclude, this exploratory study indicates that the genetic variant rs7799039 in the *LEP* gene is not associated with fat mass-related parameters in young Slovak adults. An association was observed between this variant and total body mineral estimates in women, suggesting that leptin-related genetic variability may be associated with small differences in mineral-related body composition estimates at the population level, without implying biological specificity or clinical relevance. These findings underscore the need for further investigation of genetic–nutritional interrelationships in well-characterized cohorts using more precise phenotyping approaches, to clarify their potential relevance for preventive public health strategies.

## Figures and Tables

**Table 1:** Baseline characteristics of the study sample.

Total Sample		
Age, years	22.35 ± 2.28 SD	
	**N**	**%**
**Sex**		
Women	157	50.65
Men	153	49.35
**Smoking status**		
Current smokers	59	19.03
Non-smokers	251	80.97
**Alcohol consumption**		
Daily	1	0.32
5–6 days per week	2	0.65
3–4 days per week	28	9.03
1–2 days per week	97	31.29
1–3 days per month	107	34.52
Less than once a month	66	21.29
Never	26	8.39
**Physical activity**		
Tier 0	167	53.87
Tier 1	57	18.39
Tier 2	86	27.74
**Calcium intake**		
Yes	36	11.61
No	274	88.39
**Vitamin D intake**		
Yes	52	16.77
No	258	83.23
***LEP* rs7799039 genotype and allele frequencies**		
GG	46	14.84
GA	168	54.19
AA	96	30.97
G	214	42
A	264	58

Note: N, number of participants; SD, Standard Deviation; *p*, value of statistical significance; tier 0 comprised individuals below the recommended activity levels (<300 min moderate or <150 min vigorous per week), engaging mainly in incidental movement; tier 1 included those meeting or modestly exceeding these thresholds but active only one to two times weekly; tier 2 referred to regularly active individuals surpassing 300 min of moderate or 150 min of vigorous exercise weekly, typically training three times per week.

**Table 2:** Selected anthropometric and body composition characteristics according to the *LEP* rs7799039 genotypes of the study sample.

	Women						Men					
	*LEP* GG + GA Genotype (N = 106)		*LEP* AA Genotype (N = 51)		*p*	Adjusted *p*	*LEP* GG + GA Genotype (N = 108)		*LEP* AA Genotype (N = 45)		*p*	Adjusted *p*
	Mean	SD	Mean	SD			Mean		Mean	SD		
**Weight (kg)**	60.55	10.90	62.89	13.09	0.263 ^b^	1.000	81.11	1.000	78.56	11.28	0.251 ^a^	1.000
**Height (cm)**	166.43	6.38	167.14	6.62	0.518 ^a^	1.000	180.82	1.000	179.92	7.46	0.489 ^a^	1.000
**BMI (kg/m^2^)**	21.86	3.82	22.50	4.24	0.257 ^b^	1.000	24.79	1.000	24.20	2.65	0.587 ^b^	1.000
**WHR**	0.84	0.07	0.85	0.07	0.467 ^b^	1.000	0.86	1.000	0.86	0.05	0.499 ^b^	1.000
**PBF (%)**	27.67	7.06	27.86	8.62	0.979 ^b^	1.000	18.35	1.000	18.16	4.79	0.701 ^b^	1.000
**FM (kg)**	17.42	7.82	18.30	10.01	0.764 ^b^	1.000	15.51	1.000	14.40	4.71	0.870 ^b^	1.000
**FM arm (%)**	124.36	79.86	135.58	110.47	0.937 ^b^	1.000	136.91	1.000	118.24	57.25	0.597 ^b^	1.000
**FM leg (%)**	108.97	42.80	114.65	51.67	0.727 ^b^	1.000	121.62	1.000	115.59	32.01	0.696 ^b^	1.000
**FM trunk (%)**	150.26	72.17	156.29	82.04	0.899 ^b^	1.000	177.33	1.000	167.34	61.99	0.796 ^b^	1.000
**FMI**	6.31	2.87	6.57	3.50	0.896 ^b^	1.000	4.76	1.000	4.46	1.46	0.755 ^b^	1.000
**FFM (kg)**	43.13	4.61	44.59	5.86	0.092 ^a^	1.000	65.53	1.000	64.17	9.15	0.358 ^a^	1.000
**FFMI**	15.56	1.30	15.93	1.34	0.035 ^b^	0.003 *	20.03	1.93	19.74	1.86	0.397 ^a^	1.000
**LBM (kg)**	40.55	4.32	41.90	5.54	0.096 ^a^	1.000	61.89	7.48	60.44	8.56	0.298 ^a^	1.000
**LBM arm (%)**	95.24	8.27	95.66	9.16	0.774 ^a^	1.000	104.80	10.30	102.02	10.66	0.134 ^a^	1.000
**LBM leg (%)**	103.62	7.92	103.53	8.05	0.944 ^a^	1.000	105.76	6.05	104.54	6.43	0.273 ^b^	1.000
**LBM trunk (%)**	98.65	5.01	98.15	5.27	0.562 ^a^	1.000	102.60	6.30	100.94	6.06	0.133 ^a^	1.000
**SMM (kg)**	23.52	2.73	24.37	3.54	0.099 ^a^	1.000	37.26	4.76	36.42	5.44	0.342 ^a^	1.000
**Total body** **minerals (kg)**	3.09	0.36	3.26	0.52	0.014 ^a^	0.002 *	4.56	0.62	4.51	0.73	0.600 ^b^	1.000
**Visceral FM (cm^2^)**	76.41	40.19	80.66	49.77	0.854 ^b^	1.000	65.15	39.10	60.19	23.02	0.903 ^b^	1.000
**BCM (kg)**	28.02	3.00	28.96	3.89	0.097 ^a^	1.000	43.16	5.23	42.19	5.98	0.321 ^a^	1.000
**Phase (°)**	5.18	0.43	5.25	0.57	0.467 ^b^	1.000	6.38	0.67	6.36	0.48	0.863 ^a^	1.000

Note: * Marks statistical significance difference a Holm-Bonferroni correction; ^a^ Indicates normal data distribution according to Kolmogorov–Smirnov. ^b^ Indicates non-normal data distribution according to Kolmogorov–Smirnov. Abbreviations: *LEP*, leptin; SD, Standard Deviation; BMI, Body Mass Index; WHR, Waist to Hip Ratio; PBF, Percent Body Fat; FM, Fat Mass; FFM, Fat-Free Mass; FMI, Fat Mass Index; FFMI, Fat-Free Mass Index; LBM, Lean Body Mass; SMM, Skeletal Muscle Mass; BCM, Body Cell Mass; arm %, leg %, trunk % = segmental proportion of total fat/lean mass relative to whole-body value, as provided by InBody.

**Table 3:** Analysis of selected predictors with total body minerals in women using the forward method of linear regression.

Dependent Variable	Predictors	Unstandardized B	Standardized β	95% CI for B	SE for B	*p*	R^2^	Adjusted R^2^	Durbin-Watson	T
Women										
**Total body minerals (kg)**	*LEP* AA vs. GG + GA genotype	0.133	0.147	0.027–0.239	0.054	0.015 *	0.471	0.458	1.674	0.989
	Vitamin D	0.159	0.122	0.311–0.007	0.077	0.041 *				0.989
	BMI	0.227	0.376	0.156–0.298	0.036	<0.001 *				0.986
	Height	0.036	0.550	0.029–0.044	0.004	<0.001 *				0.981
	The least significant variables: smoking status, alcohol consumption, physical activity, calcium intake									

Note: * Marks statistical significance *p* < 0.05. Abbreviations: B, beta coefficient; CI, Confidence Interval; *p*, value of statistical significance (linear regression analysis, forward method); R^2^, coefficient of determination; SE, Standard Error; T, tolerance (collinearity statistics).

## Data Availability

The data presented in this study are available on request from the corresponding author. The data are not publicly available due to ethical restrictions and the inclusion of potentially identifiable personal data.
